# CB_2_ cannabinoid receptor activation promotes colon cancer progression via AKT/GSK3β signaling pathway

**DOI:** 10.18632/oncotarget.11968

**Published:** 2016-09-12

**Authors:** Esther Martínez-Martínez, Asunción Martín-Ruiz, Paloma Martín, Virginia Calvo, Mariano Provencio, José M. García

**Affiliations:** ^1^ Department of Medical Oncology, Hospital Universitario Puerta de Hierro-Majadahonda, E-28222 Madrid, Spain; ^2^ Department of Pathology, Hospital Universitario Puerta de Hierro-Majadahonda, E-28222 Madrid, Spain

**Keywords:** CB_2_, colon cancer, AKT/PKB, JWH-133, proliferation

## Abstract

The pharmacological activation of the cannabinoid receptor type 2, CB_2_, has been shown to elicit anti-tumoral mechanisms in different cancer types. However, little is known about its endogenous role in tumor pathophysiology, and different studies have attributed pro-tumorigenic properties to this receptor. In a previous work, we showed that CB_2_ expression is a poor prognostic factor in colon cancer patients. Here we report that activation of CB_2_ with low doses of specific agonists induce cell proliferation and favor the acquisition of aggressive molecular features in colon cancer cells. We show that sub-micromolar concentrations of CB_2_-specific agonists, JWH-133 and HU-308, promote an increase in cell proliferation rate through the activation of AKT/PKB pathway in colon cancer *in vitro* and *in vivo*. AKT activation promotes GSK3β inhibition and thus, a more aggressive cell phenotype with the subsequent elevation of SNAIL levels, E-cadherin degradation and β-catenin delocalization from cell membrane. Taken together, our data show that CB_2_ activation with sub-micromolar doses of agonists, which could be more similar to endogenous levels of cannabinoids, promote colon cancer progression, implicating that CB_2_ could have a pro-tumorigenic endogenous role in colon cancer.

## INTRODUCTION

Based on incidence and mortality, colon cancer is the second leading cause of cancer death in developed countries and the fourth in the world, with more than one million new cases of colon cancer diagnosed each year [1, 2]. Despite considerable progress in recent decades in surgical oncology and the application of new radiation and chemotherapy treatments, mortality by colon cancer remains high due to metastasis and resistance to current treatments. Thus, understanding the mechanisms involved in the control of tumor growth and the development of chemopreventive agents and targeted therapies are major goals of basic research in oncology.

Cannabinoids, the active compounds obtained from *Cannabis sativa* and their derivatives, have been primarily used with palliative purposes in cancer patients. However, in recent years, those compounds (plant-derived or synthetically-produced) have been proposed for their use as anticancer agents since different studies have attributed them anti-tumoral effects such as induction of apoptosis, cell cycle arrest or inhibition of cell migration and angiogenesis [3, 4]. Cannabinoids exert their effects through the endogenous system called endocannabinoid system (ECS). ECS is comprised of two major cannabinoid-specific receptors, CB_1_ and CB_2_; along with the endocannabinoids anandamide (AEA) [5] and 2-arachidonoylglycerol (2-AG) [6, 7] and the enzymes that carry out their biosynthesis and degradation. There exist other receptors that have also been proposed as endocannabinoid receptors, the transient receptor potential cation channel subfamily V member 1 (TRPV1) or the orphan G protein-coupled receptor (GPR55) [8]. The CB_1_ receptor is mainly present in the central nervous system and so mediates the psychotropic effects of exogenous cannabinoids, whereas the CB_2_ receptor is mainly expressed in peripheral and inflammatory tissues [9]. It is described that the ECS undergoes adaptive changes during tumor development, which are in general an increase in endocannabinoid and cannabinoid receptors expression levels and a decrease in the levels of the enzymes responsible for endocannabinoid degradation (i.e. FAAH), although there can be some exceptions specific to tumor types [4]. Specifically for colorectal cancer, an increase in endocannabinoid levels, a down-regulation of CB_1_ and an up-regulation of CB_2_ receptor expression is described [10–12].

Several evidences points to the cannabinoid receptor CB_2_ as a target for anti-tumoral therapy in several types of cancer [3, 4, 12–14], but little is known about its role in tumor generation and progression. In fact, a contradictory role for the ECS in cancer pathophysiology is currently being discussed, with some works reporting anti-tumoral effects of cannabinoids, and others pointing to a possible tumor-promoting and immunosuppressive role [15–20]. Besides, some reports show that the EC_50_ values of exogenous administered cannabinoid drugs, usually in the micromolar range, may not reflect the endogenously produced endocannabinoids levels, which reach at most nanomolar concentration locally [21]; therefore physiological effects could differ from pharmacological ones [15, 21]. Moreover, Hart *et al.* demonstrated that THC levels comparable to those found in serum of patients that underwent THC treatment accelerated the proliferation of cancer cells [20]. This suggests that cannabinoids action on tumor progression is strongly dose-dependent, and a bimodal action it is possible with low (endo)cannabinoid levels being pro-proliferative and high doses of exogenous agonists being anti-proliferative and pro-apoptotic [15]. Thus, a deeper knowledge of the physiological role of CB_2_ in the relevant processes of cancer development, such as cell survival and proliferation, is necessary before proposing CB_2_ agonists as anti-tumoral drugs.

The aim of this study was to investigate on *in vitro* and *in vivo* murine model whether CB_2_ receptor activation with a wide range of doses of agonist results in pro-tumoral effects and, in this case, to identify the possible molecular mechanisms underlying this oncogenic action. In particular, we explored the hypothesis that cannabinoid receptor activation might induce pro-proliferative effects through the activation of PI3K/AKT survival pathway.

## RESULTS

### Biphasic effect of synthetic cannabinoids, JWH-133 and HU-308, on proliferation of colon cancer cells

We first analyzed the effect of different doses of specific synthetic CB_2_ agonists on cell proliferation with the colon cancer derived cell line HT29. Cells were incubated in the presence of increasing concentrations of JWH-133 or HU-308 for 48h, and cell viability was evaluated by CCK-8 viability assay and by flow cytometry.

**Figure 1:**
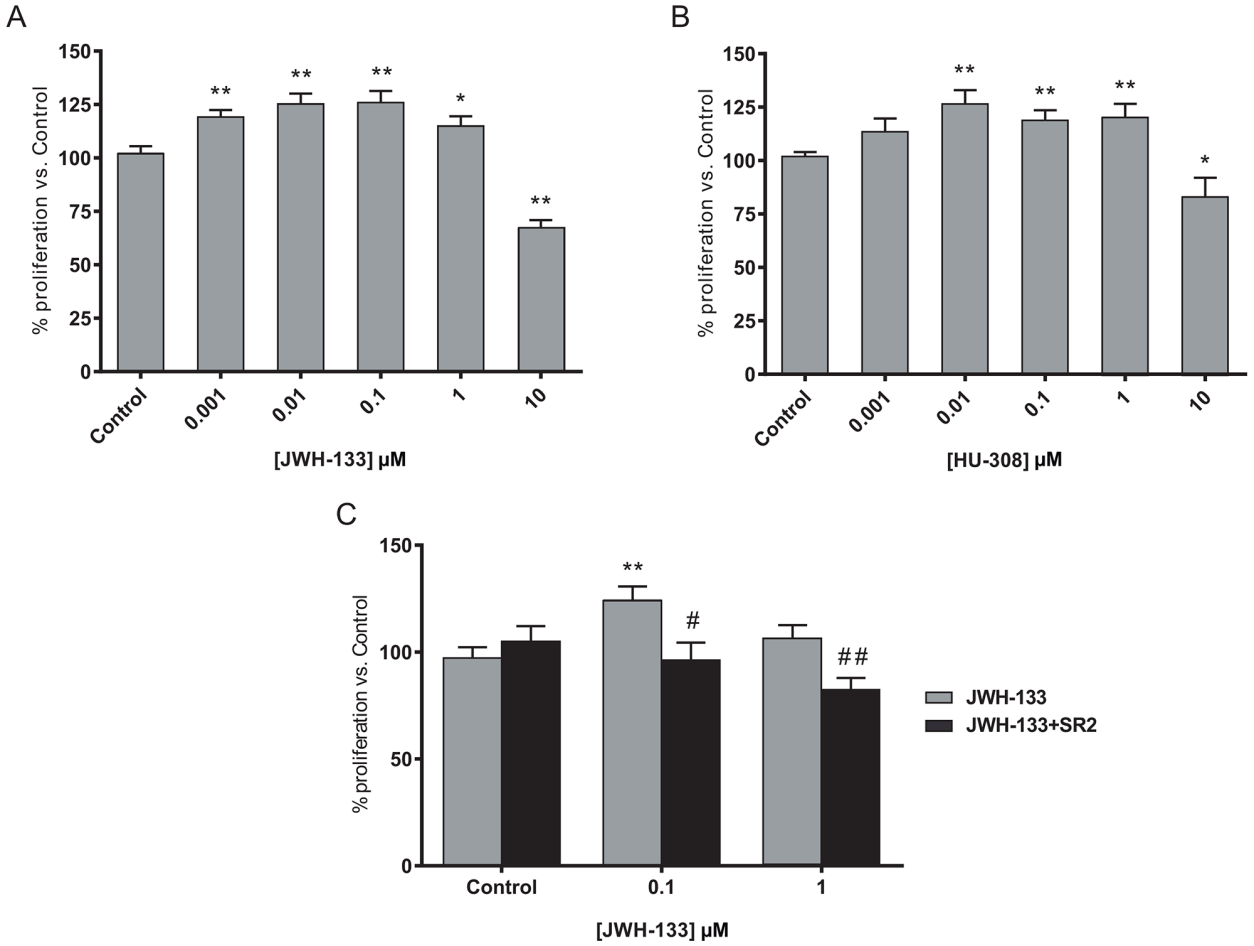
Biphasic effect of CB_2_ agonists on colon cancer cell line **A.** HT29 cells were incubated with increasing concentrations of JWH-133 or **B.** HU-308 for 48h and cell viability was assayed with CCK-8. **C.** HT29 cells were incubated with 0.1 or 1 μM JWH-133 for 48h in the presence or absence of 0.5 μM CB_2_ antagonist, SR 144528 (SR2). Control JWH-133 group, DMSO; Control in JWH-133+SR2, 0.5 μM SR2. Data are the means ± s.e. of two different experiments, each performed with six replicates. *p < 0.05 and **p < 0.01 using Student's t-test for the comparison between vehicle-treated and cannabinoid-treated cells; and #*p* < 0.05 and ##*p* < 0.01 for the comparison between cannabinoid-treated and antagonist-treated cells.

As shown in Figure 1A and 1B the proliferation increased until 1 μM concentration, while 10 μM decreased cell survival; resulting in a biphasic proliferation curve. Similar results were obtained with JWH-133 in SW480 and LS174T cell lines (Supplementary Figure S1A), verifying that it was not a cell-specific effect. However, the effects were more evident with HT29 cell line, so most of the experiments were performed with this cell line. The blockade of CB_2_ with the antagonist SR 144528 (SR2) verified that the effect is CB_2_-mediated since the increase in cell proliferation with JWH-133 0.1 or 1 μM is prevented by co-incubation with SR2 0.5 μM (Figure 1C, Supplementary Figures S1B and S1C).

**Figure 2:**
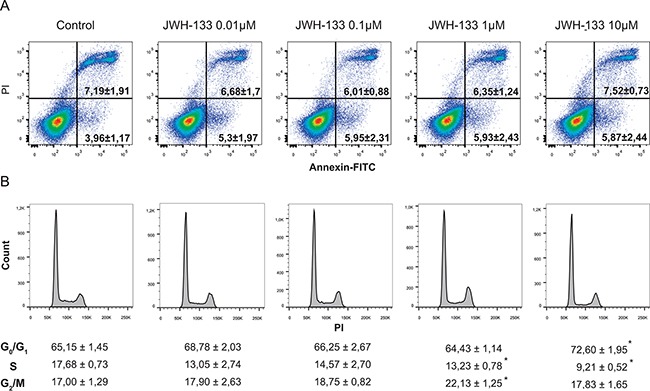
**A.** Evaluation of apoptosis by Annexin V-FITC/IP staining. Representative plots of Annexin V-FITC/IP staining of HT29 cells cultured in the presence of increasing concentrations of JWH-133 for 48h, including the percentage of early apoptotic cells (bottom right quadrant) and late apoptotic cells (upper right quadrant). **B.** Cells were incubated in the presence of increasing concentrations of JWH-133 for 48h and cell cycle was assayed by flow cytometry. Data are the means ± s.e. of three different experiments. *p < 0.05 using Student's t-test for the comparison between vehicle-treated and cannabinoid-treated cells.

To study the effect of JWH-133 on apoptosis and to discard a toxic effect of the vehicle (DMSO), we performed an Annexin V-FITC/IP staining in HT29 cells. We observed no significant differences in cell death between different doses (Figure 2A), therefore the increase in cell viability is due to an increase in proliferation and not to a reduced survival in control samples rescued by JWH-133 treatment. Besides, a cell-cycle analysis demonstrated that cannabinoid treatment resulted in a moderate, although repeated and significant accumulation of cells in G_2_/M phase (Figure 2B) at 0.1 and 1 μM of JWH-133. These results suggested that the agonist could be inducing faster progression of the cell cycle and thus an increase in cell division. Moreover, we observed that 10 μM of JWH-133 did not trigger apoptosis, since no significant changes were detected (Figure 2A), but promoted an increase of cells in G_0_/G_1_ phase (Figure 2B); which suggest that it is inducing a cytostatic, instead of a cytotoxic, effect.

Since the maximal increase in proliferation was reached at JWH-133 0.1 μM in HT29 cells, we decided to carry out the subsequent studies with this stimuli.

### PI3K/AKT signaling pathway is activated after JWH-133 treatment

It has been previously reported that G protein-coupled receptors (GPCRs), the family of receptors to which CB_2_ belongs, can stimulate the serine/threonine protein kinase AKT/PKB (Protein Kinase B) activity [22]; which is a signal transduction pathway that promotes tumorigenesis by stimulating cell cycle progression, proliferation and activating anti-apoptotic mechanisms [23]. Therefore, we hypothesized that AKT pathway may be involved in the increase on cell proliferation triggered by JWH-133.

**Figure 3:**
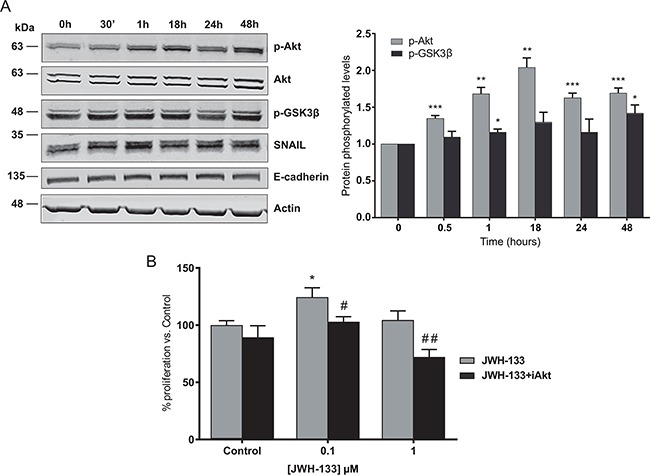
Signaling mechanisms activated by JWH-133 0.1 μM in HT29 cells **A.** Cells were incubated for different times with 0.1 μM of JWH-133 in serum-free medium. The phosphorylation of AKT and GSK3β, and SNAIL and E-cadherin levels were analyzed in the cells by western blot (WB) analysis using the indicated specific antibodies. Representative blots of three different analyses are shown (left). Densitometric analysis of AKT and GSK3β phosphorylation levels was performed with ImageJ (NIH) software and data are represented as the means ± s.e. of three different experiments. *p < 0.05, **p < 0.01 and ***p < 0.001 using Student's t-test for the comparison between vehicle-treated and cannabinoid-treated cells (right). **B.** HT29 cells were incubated with 0.1 or 1 μM JWH-133 for 48h in the presence or absence of 0.5 μM AKT inhibitor, iAKT 1/2 (iAkt) and cell viability was assayed with CCK-8. Control JWH-133 group, DMSO; Control in JWH-133+iAkt, 0.5 μM iAkt. Data are the means ± s.e. of three different experiments, each performed with six replicates. *p < 0.05 using Student's t-test for the comparison between vehicle-treated and cannabinoid-treated cells; and #*p* < 0.05 and ##*p* < 0.01 for the comparison between cannabinoid-treated and iAkt-treated cells.

As shown in Figure 3A, JWH-133 enhanced AKT phosphorylation in HT29 cells in a time dependent manner, reaching the maximal levels at 18h and 48h. The involvement of the PI3K/AKT cascade in CB_2_ agonist-mediated enhancement of cell proliferation was further tested by examining the phosphorylated levels of a downstream target, the glycogen synthase kinase-3β (GSK3β). Treatment with JWH-133 leads to AKT-mediated phosphorylation of GSK3β, which levels are increased in a time-dependent manner and reached the highest levels at 48h (Figure 3A). Additionally, we verified that AKT activation is involved in the JWH-133 induced cell proliferation, as its blockade with a specific inhibitor (iAKT) prevented the increase in cell viability in HT29 cells (Figure 3B). Similar results were obtained with SW480 and LS174T cell lines, verifying that AKT activation is involved and necessary for the pro-proliferative effects of low concentration of JWH-133 (Supplementary Figures S1B, S1C and S2).

### GSK3β inactivation through CB_2_ signaling leads to an increase in SNAIL levels

GSK3β is a serine/threonine protein kinase constitutively active in unstimulated cells, but its phosphorylation by p-AKT leads to its inactivation [23]. This kinase is not only involved in the control of cell proliferation, but it is described that it is also related with the epithelial-mesenchymal transition (EMT), the first step in the metastatic process. Concretely, GSK3β reduces the stability of SNAIL, a zinc-finger transcription factor implied on the initiation of the EMT, by inducing it degradation [24]. We observed a significant increase in SNAIL protein levels in HT29 cells stimulated with JWH-133 0.1 μM that correlated with the increment of p-AKT and p-GSK3β levels (Figure 3A)

**Figure 4:**
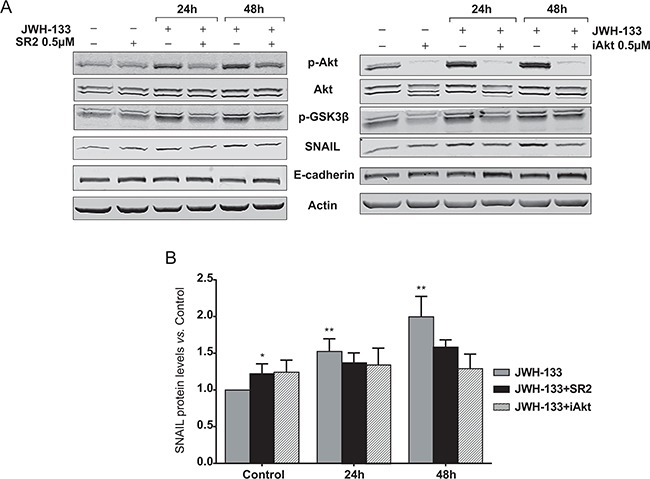
Inhibition of AKT/PKB and GSK3β phosphorylation, and SNAIL stabilization by the CB_2_ antagonist, SR2; and the AKT inhibitor, iAkt **A.** HT29 cells were pre-incubated with 0.5 μM SR2 (left panel) or 5 μM iAkt (right panel) for 5h prior to be incubated for 24h and 48h with JWH-133 0.1 μM in presence or absence of 0.5 μM SR2 or 0.5 μM iAkt. Representative blots of three different analyses are shown. **B.** Densitometric analysis of SNAIL was performed with ImageJ (NIH) software and data are represented as the means ± s.e. of three different experiments. *p < 0.05 and **p < 0.01 using Student's t-test for the comparison between vehicle-treated and cannabinoid-treated cells.

We confirmed that the increase in p-AKT, p-GSK3β and SNAIL levels were CB_2_-specific and that AKT activation is key for the downstream effects, since CB_2_ and AKT blockade with specific inhibitors (SR2 and iAKT, respectively) prevented the enhancement of p-AKT, p-GSK3β and SNAIL levels induced byJWH-133 (Figure 4 and Supplementary Figure S2).

### Membrane-bound E-cadherin loss after JWH-133 treatment and disassembly of cell-cell contacts

Since SNAIL stabilization could suggest the initiation of EMT, we proceeded to study the state of the cell-cell interactions, which are primarily affected in this process. E-cadherin is one of the most important molecules in the maintenance of cell interactions by forming the adherens junctions (AJs) in the cell surface. Due to SNAIL is a well-described repressor of E-cadherin transcription, we evaluated mRNA levels of E-cadherin, but we did not find differences due to JWH-133 treatment (data not shown). However, we evaluated changes in protein by western blot, and we found a slight but repeated decrease in E-cadherin levels at 48h (Figure 3A), that was prevented by the blockade of CB_2_ and AKT with the specific inhibitors (Figure 4A).

**Figure 5:**
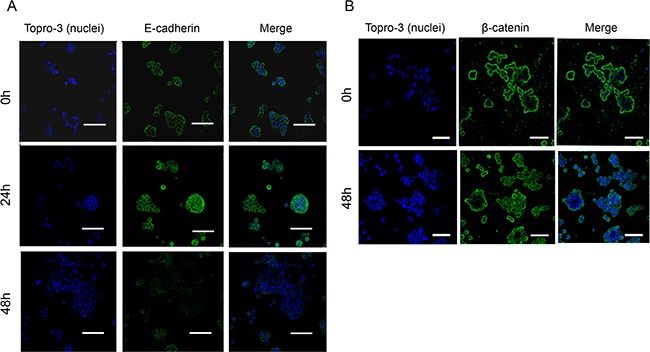
Regulation of E-cadherin and β-catenin localization and stability by JWH-133 0.1 μM treatment HT29 cells were incubated with JWH-133 0.1 μM for 24h and/or 48h in serum-free medium. Cells were stained with specific **A.** E-cadherin (green) or **B.** β-catenin (green) antibodies and photographed with confocal microscopy. Nuclei were stained with Topro-3 (blue). Scale bars, (**A**) 55μm and (**B**) 50μm. Representative image of three different experiments.

As E-cadherin has to be membrane-attached to maintain cell-cell interactions, we analyzed by confocal microscopy the localization of this protein after treatment with JWH-133 0.1 μM. It can be observed in Figure 5A that E-cadherin remains unaffected in control, showing membrane location; while after 24h of treatment suffers a delocalization from membrane to cytoplasm. After 48h of treatment, E-cadherin has nearly disappeared from the whole cell cluster in many of the analyzed fields. This could suggest that E-cadherin could be suffering some post-transcriptional modification that induce its disassembling from cell surface and its posterior degradation, but the levels of SNAIL, although are elevated, might not be enough to trigger E-cadherin transcriptional repression.

In AJs E-cadherin binds through its cytoplasmic domain to different proteins, one of them β-catenin. Upon E-cadherin degradation, β-catenin remains free in cytoplasm and, in normal conditions, it is degraded by a complex that includes GSK3β. However, if β-catenin cannot be degraded it is transported to the nucleus where it exerts a signaling function by promoting the transcription of genes related with cell proliferation [25, 26]. As we have seen E-cadherin degradation and GSK3β inactivation, we decided to investigate whether β-catenin is transported to the nucleus where it would promote the transcription of genes that contribute to cell proliferation. By confocal microscopy we observed that after 48h of treatment with JWH-133 at 0.1 μM, in accordance with E-cadherin degradation, β-catenin is internalized from the membrane into the cytoplasm and nucleus, without changes in total levels (Figure 5B).

Altogether, these data suggest that CB_2_ stimulation with sub-micromolar doses of the agonist lead to the activation of PI3K/AKT signaling axis, and to the increase in tumor cell proliferation and aggressiveness.

### Differential response of colon cancer xenografts to diverse doses of JWH-133 in nude mice

**Figure 6:**
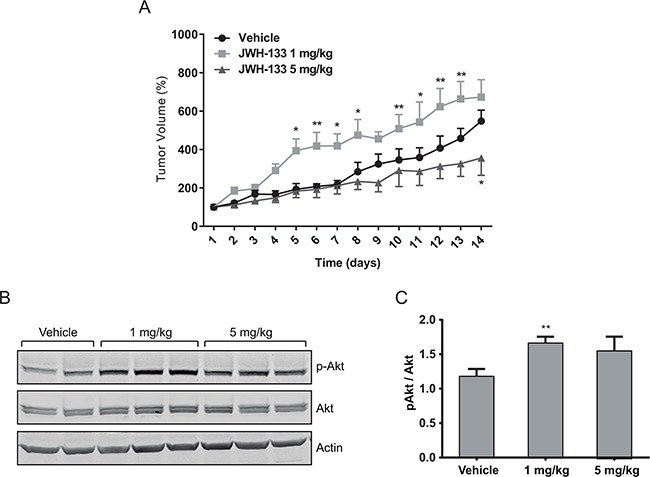
*In vivo* effects of JWH-133 Nude mice were injected s.c. in the right flank with HT29 cells and a week later (day 1) were treated for 14 days with vehicle (control, DMSO), 1 mg/kg JWH-133 or 5 mg/kg JWH-133. Treatments were carried out by intraperitoneal injections every day. Tumor volumes were measured daily. **A.** Tumor growth curve after administration of vehicle (circles), 1 mg/kg JWH-133 (squares) or 5 mg/kg JWH-133 (triangles). Results represent the mean ± s.e. of five mice in each group. *p < 0.05 and **p < 0.01 using two-way ANOVA with a Student-Newman-Keuls test post hoc analysis. **B.** Western blot analysis and **C.** densitometric analysis of phospho-AKT in tumors from the three different mice groups. Results represent the mean±s.e. of p-AKT levels of five mice in each group, **p < 0.01 using Student's t-test for the comparison between vehicle-treated and cannabinoid-treated groups.

To asses our hypothesis in an *in vivo* model, subcutaneous tumors were generated in nude mice with HT29 cells. Tumor-bearing animals were treated daily with the vehicle, 1 mg/kg JWH-133 or 5 mg/kg JWH-133 for 14 days. Tumor volume was calculated every day, and at the end of the experiment tumors were dissected. As shown in Figure 6A, tumors increased their growth rate significantly in response to 1 mg/kg of JWH-133 with respect to the vehicle-treated group; whereas a 5 mg/kg of JWH-133 produced the opposite effect, a reduction in tumor growth rate (although significant effect is only observed at day 14).

In agreement to our *in vitro* results, we observed significant higher p-AKT levels in the 1 mg/kg-treated group respect to the vehicle- or 5 mg/kg-treated groups (Figure 6B). However, in the 5 mg/kg-treated group p-AKT levels are higher (although not significant) than in the vehicle-treated one, which could be because the bioavailability of JWH-133 may vary in different areas of the tumor, leading to intra-tumoral variability of the effect.

## DISCUSSION

Despite the controversy about the role of the endocannabinoid system (ECS) in tumor generation and progression, there is increasing evidence that shows that the ECS might play a pro-tumorigenic role [4, 15, 18, 28, 29]. Indeed, in a previous study we demonstrated that CB_2_ expression correlates with worse overall and disease free survival in colon cancer patients, setting it as a poor prognostic factor for those patients [30]. Here we show that the activation of the CB_2_ receptor with doses of exogenous agonists, JWH-133 and HU-308, below the micromolar range promotes cell proliferation in colon cancer derived cell lines. We observed a biphasic effect on proliferation of HT29, SW480 and LS174T where agonist concentrations below 1 μM promote cell growth while a 10 μM dose is necessary to produce cytostatic effects by arresting cell cycle in G_0_/G_1_ phase. It has been recently described that the bimodal response to cannabinoids through CB_2_ receptor can be due to the existence of CB_2_-GPR55 heteromers, and that low doses of agonists (THC, in this case) activate CB_2_ exclusively, leading to pro-tumoral actions; but higher doses of THC also binds to GPR55 and exerts a cross-antagonism that inhibits CB_2_ pro-proliferative signaling [27]. It can be interesting in future studies to analyze the presence of CB_2_-GPR55 heteromers in our colon cancer model to shed light on the mechanisms underlying the biphasic effect of CB_2_ agonists.

As the molecular mechanisms underlying both pro- and anti-tumoral effects of CB_2_ activation are not well-described yet, we decided to study in depth those mechanisms. We found that JWH-133, with a dose that has previously shown to promote the highest proliferation levels in HT29, increased AKT phosphorylation in a CB_2_-specific manner. As a consequence, p-AKT phosphorylated the glycogen synthase kinase-3β (GSK3β), leading to its inactivation. GSK3β is constitutively active in unstimulated cells and acts as a “tumor suppressor” since maintains in inactivate state different molecules related to cell growth, such as cyclin D1 or c-Myc [23, 31]. GSK3β is also involved in SNAIL degradation [32], so as a consequence of its inactivation after JWH-133 treatment, we observed an increase in SNAIL protein levels. Despite SNAIL is a transcriptional repressor of E-cadherin we did not find a decrease in their mRNA levels in response to increased levels of SNAIL. However, since we observed decrease of E-cadherin protein levels and its delocalization from the membrane, we hypothesized that these effects might be mediated by a post-transcriptional regulation that finally destabilize the cell-cell interactions [25]. These results could be in agreement with previous studies showing that upon activation of Src tyrosine kinase, which could occur during EMT, adherens junctions are destabilized due to E-cadherin phosphorylation, which is then internalized and finally degraded in the lysosome [25, 33]. Further studies are necessary to unveil the mechanism that leads to E-cadherin degradation after CB_2_ activation with low doses of agonists. Nevertheless, the destabilization of E-cadherin from membrane was verified because β-catenin was released from cell membrane and internalized into the intracellular and nuclear space. In normal conditions, the released β-catenin would be degraded through a complex that involves the adenomatous polyposis coli (APC)-complex consisting of APC, axin, diversin, casein kinase I, and GSK3β. However, the lack of function of any of these components, such as GSK3β, prevents β-catenin degradation [34]. In these conditions, β-catenin is transported to the cell nucleus where it binds to T-cell factor/lymphoid enhancer factor (TCF/LEF) DNA binding proteins, which regulate transcription of genes such as c-Myc or cyclin D1 [35]. Since GSK3β is inhibited due to JWH-133 stimulation, the released β-catenin, instead of being degraded, is internalized into the cytoplasm and nucleus, where it could be exerting pro-tumoral actions. Besides, β-catenin stabilization is also influenced because the HT29 cell line possess a truncated form of APC, which is a typical characteristic in colon cancer, and it is only partially active; so the degradation of the free cytoplasmic β-catenin is less efficient compared to cells possessing the wild type form of this protein [36].

Finally, our *in vivo* model further confirmed that the effect on cell proliferation is strongly dependent on cannabinoid concentrations. While a dose of 5 mg/kg of JWH-133 decreased the tumor proliferation rate, a dose of 1 mg/kg significantly increased it. Moreover, in agreement with the previous *in* vitro study, the group with the higher proliferation rate has greater levels of p-AKT. This strengthens our hypothesis that CB_2_ activation with a low dose range of agonists leads to an increase of proliferation and pro-tumoral effect by activation of PI3K/AKT signaling pathway.

In conclusion, the present study shed light on the physiological contribution of CB_2_ to cell functions relevant for cancer progression, since the activation of CB_2_ with doses that are not capable of triggering apoptosis, below the micromolar range, increases the proliferation and aggressiveness on colon cancer cell lines. These results have to be taken into account when a cannabinoid-based therapy is being considered for colon cancer patients due to the concentration-dependent response and the difficulty for the tumor-site delivery of cannabinoid-derived drugs.

## MATERIALS AND METHODS

### Reagents and drugs

JWH-133 and HU-308 were purchased from Tocris Cookson (Bristol, UK), and the CB_2_ antagonist SR 144528 was from Cayman Chemical (Ann Arbor, MI). The drugs were dissolved in dimethylsulfoxide (DMSO). Antibody anti-phospho-AKT (Ser473), anti-total-AKT and anti-phospho-GSK3β (Ser9) were obtained from Cell Signaling Technology (St Louis, MO, USA). We purchased antibodies anti-SNAIL, anti-β-Actin, and AKT Inhibitor (AKTi-1/2) from Abcam (Cambridge, UK). Anti-E-cadherin antibody is from Becton Dickinson (San Jose, CA, USA) and anti-β-catenin antibody is from Dako (Glostrup, Denmark).

### Cell culture

Human colon carcinoma cells HT29, SW480 and LS174T were purchased from American Type Culture Collection (Rockville, MD, USA) and were cultivated Dulbecco's modified Eagle medium (DMEM) (Gibco Life Technologies, Gergy-Pontoise, France), containing 10% heat-inactivated fetal calf serum (FCS), 2mM L-glutamine, penicillin (100 U/mL), streptomycin (100 ng/mL) and fungizone (0.25 μg/mL) at 37°C in a 5% CO_2_-humidified atmosphere. The cell lines were authenticated by STR DNA profiling analysis. Cells were seeded sub-confluently and, before the experiments, the serum was removed overnight. For the studies with the CB_2_ antagonist (SR2) and AKT inhibitor (iAKT), cells we pre-incubated with SR2 0.5 μM or iAKT 5 μM alone for 5h, after that the medium was replaced with medium containing JWH-133 at the indicated concentration with 0.5 μM SR2 or 0.5 μM iAKT.

### Cell viability assays

Cells were set up 4x10^3^ cells per well of a 96-well plate and were cultured in DMEM medium supplemented with 10% FCS overnight. The medium was replaced for serum-free DMEM medium and cells were incubated for 48h with the treatments according to figure legends. Cell viability was assayed by Cell Counting Kit-8 (CCK-8) (Dojindo EU GmbH, Munich, Germany) according to the manufacturer's protocol.

### Flow cytometry

Flow cytometry was used to detect apoptotic cells and the distribution of cell cycle. 3x10^5^ cells were seeded per well of a 6-well plate. After being cultivated with medium alone or medium containing the indicated stimuli for 48h, cells were harvested. To analyze apoptosis by Annexin V/PI (Propidium Iodure) staining, the cells were washed twice with PBS and incubated in 0.1 ml of binding buffer 1x with 5μL Annexin V-FITC for 15 min. Then 5μL of PI were added and finally cells were suspended in a final volume of 0.3 ml of binding buffer 1x. For cell cycle distribution analysis, the cells were washed twice with PBS and fixed overnight at 4°C with cold 100% ethanol. After eliminating and washing ethanol, cells were incubated overnight in 0.3 ml of PBS with 0.2 mg/ml Rnase and 0.02 mg/ml PI. In both, 20 000 cells of each sample were analyzed by flow cytometry in a MACSQuant (Miltenyi Biotec, Bergisch Gladbach, Germany).

### Western blot analysis

Cells were cultured at 3x10^5^ cells per well of a 6-well plate and incubated with the treatment and times described in the Results section. Cells were lysed into T-PER buffer supplemented with proteases and phosphatases inhibitors 1x. Protein concentration was determined using the Pierce BCA protein assay kit (Life Technologies, Carlsbad, CA). Proteins were separated in SDS-PAGE and blotted on Nitrocellulose membranes. Blots were incubated overnight in 4°C with the following antibodies: anti-E-cadherin (1/1500), anti-phospho-AKT (1/2000), anti-total-AKT (1/1000), anti-phospho-GSK3β (1/1000), anti-SNAIL (1/500) and anti-β-Actin (1/2000) as loading control. The next day, blots were incubated 1 hour at room temperature with goat anti-rabbit or anti-mouse IRDye800^®^ and IRDye700DX^®^ conjugated secondary antibodies (Rockland, Limerick, PA). Densitometric analysis was done with Image-J (NIH).

### Confocal microscopy

Cells were set up 5x10^4^ cells per well of a 4-well Nunc™ Lab-Tek™ II Chamber Slide™ System (Thermoscientific, Waltham, MA) and treated for 24h and 48h with JWH-133 0.1 μM, when necessary. After treatment, cells were fixed with Methanol for 10 minutes, washed with PBS, incubated in 50 mM NH_4_Cl and blocked with 5% BSA to reduce non-specific protein binding. Cells were incubated with Anti E-Cadherin (1/25) or Anti β-catenin (ready-to-use) overnight at 4°C, washed with PBS and followed with Alexa Fluor 488 anti-mouse (Invitrogen Life Technologies, 1/1000) for 45 minutes at room temperature. Nuclei were stained with Topro-3 (Invitrogen Life Technologies, 1/500) for 20 minutes. The chambers were removed from the glass slide and cells were visualized with inverted Microscopy. Images of the specimens were collected with a TCS SP5 confocal microscope (Leica Microsystems, Wetzlar, Germany), equipped with 10×0.22 and at an optical zoom of 3. Z-series images were obtained through the collection of serial, confocal sections at 1 μm intervals.

### *In vivo* xenograft model

All animal studies were conducted in accordance with the Spanish institutional regulation for the housing, care and use of experimental animals, have been carried out with ethical committee approval and met the European Community directives regulating animal research. Recommendations made by the UKCCCR have been adhered to carefully. Swiss nude (nu/nu) 5-week-old female mice were purchased from Charles River (Chatillon-sur-Chalaronne, France) and were housed in a laminar airflow cabinet under pathogen-free conditions on a 12-h light–dark schedule. Mice were injected subcutaneously (s.c.) in the right flank with 5x10^6^ HT29 cells in 0.1 ml of PBS. One week after transplantation, mice were divided into three experimental groups of five animals each, which received the following treatments as intraperitoneal (i.p.) injections: group 1, 5% DMSO (control); group 2, 1 mg/kg body weight (b.w.) JWH-133; group 3, 5 mg/kg b.w. JWH-133. The injection was repeated every day and treatment was continued for 14 days. Tumor volumes were monitored every day using calliper measurements and were calculated using the following formula: (4π/3) × (*w*/2)^2^ × (*l*/2), where *w* = width and *l* = length. At the end of the treatment, animals were sacrificed, and tumors were dissected and divided into pieces, which were cryoembedded in cryo-preservative O.C.T compound (Tissue-Tek) or paraffin-embedded for further analysis. Protein extraction from dissected sample was performed with T-PER buffer supplemented with proteases and phosphatases inhibitors 1x (1g tissue/10 mL buffer) as above described.

### Statistical analysis

Data are presented as mean ± s.e. (standard error) of the number of experiments indicated. Statistical comparisons among groups were made with Student's t-test, and the difference was considered to be statistically significant when the *p*-value was < 0.05.

Tumor growth curves in mice xenograft model were compared by two-way ANOVA with a post hoc analysis by Student-Newman-Keuls test with Graph-Pad Prism 6 software.

## SUPPLEMENTARY MATERIALS FIGURES


