# Supplementary material for: CB2 cannabinoid receptor activation promotes colon cancer progression via AKT/GSK3β signaling pathway
Source: Oncotarget. 2016 Sep 12;7(42):68781–91. doi: 10.18632/oncotarget.11968 (PMC5356589; doi:10.18632/oncotarget.11968)
Supplement: Supplementary file 1 [file oncotarget-07-68781-s001.pdf]

CB_2 cannabinoid receptor activation promotes colon cancer progression via AKT/GSK3β signaling pathway

SUPPLEMENTARY FIGURES

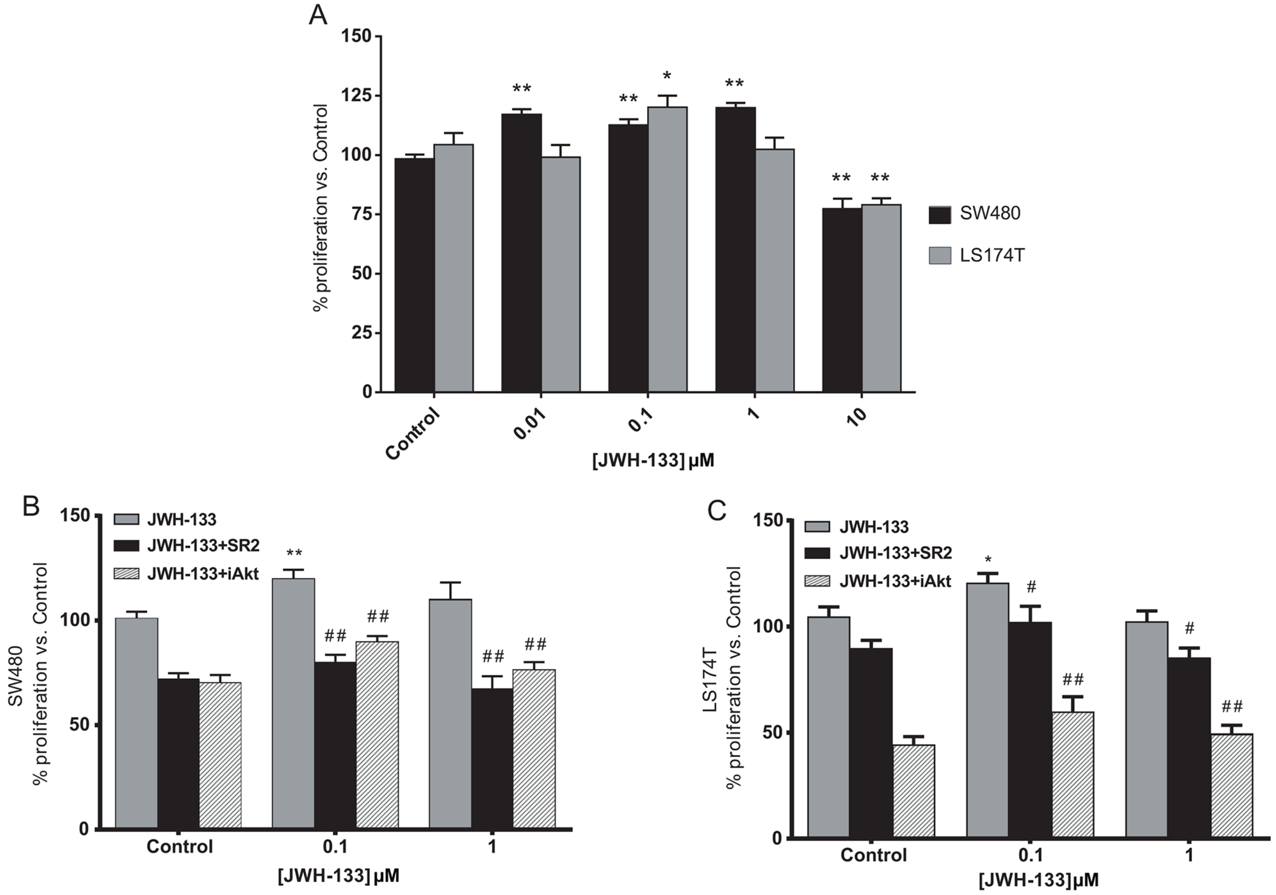

Supplementary Figure S1: Biphasic effect of CB_2 agonists on colon cancer cell lines. A. SW480 and LS174T cells were incubated with increasing concentrations of JWH-133 for 48h and cell viability was assayed with CCK-8. **B.** SW480 and **C.** LS174T cells were incubated with 0.1 or 1 µM JWH-133 for 48h in the presence or absence of 0.5 µM SR2 or 0.5 µM iAkt. Control JWH-133 group, DMSO; Control in JWH-133+SR2 group, 0.5 µM SR2; Control in JWH-133+iAkt group, 0.5 µM iAkt. Data are the means ± s.e. of two different experiments, each performed with six replicates. *p < 0.05 and **p < 0.01 using Student's t-test for the comparison between vehicle-treated and cannabinoid-treated cells; and #p < 0.05 and ##p < 0.01 for the comparison between cannabinoid-treated and antagonist and inhibitor-treated cells.

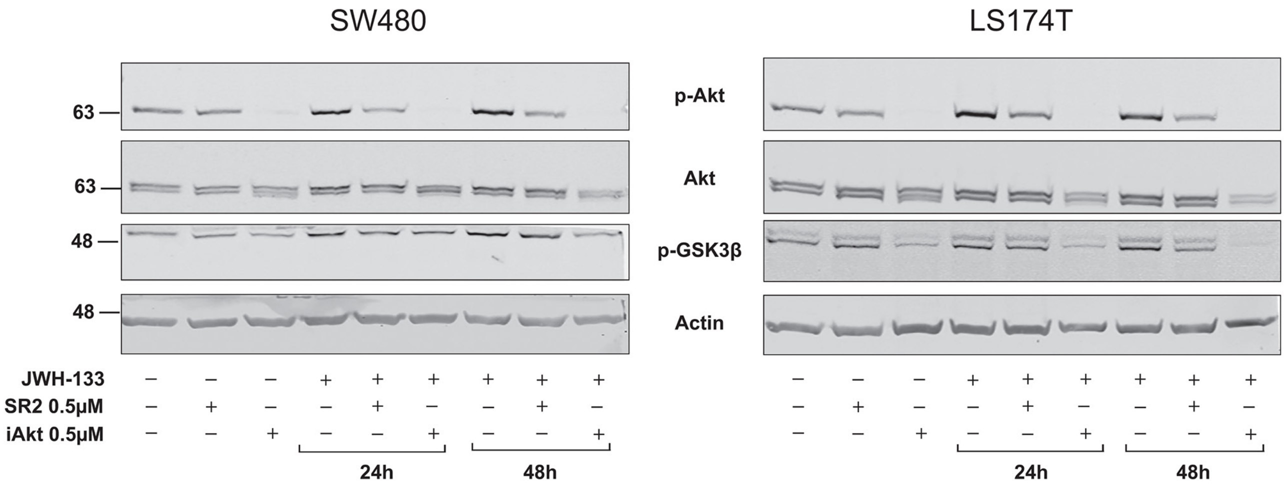

Supplementary Figure S2: CB_2-mediated activation of AKT pathway in SW480 and LS174T cell lines. SW480 (left panel) and LS174T (right panel) were pre-incubated with 0.5 µM SR2 or 5 µM iAkt for 5h prior to be incubated for 24h and 48h with JWH-133 0.1 µM in presence or absence of 0.5 µM SR2 or 0.5 µM iAkt. The phosphorylation of AKT and GSK3β was analyzed in the cells by WB analysis using the indicated specific antibodies. Representative blots of three different analyses are shown.
